# *Nitrospira* dominant pin-point flocs with granule-like settleability in stirred tank reactors with oxic/hypoxic/oxic zones

**DOI:** 10.3389/fmicb.2023.1307727

**Published:** 2023-12-04

**Authors:** Hussain Aqeel, Bruke Asefa, Steven N. Liss

**Affiliations:** ^1^Chemistry and Biology, Toronto Metropolitan University, Toronto, ON, Canada; ^2^School of Environmental Studies, Queen’s University, Kingston, ON, Canada; ^3^Department of Microbiology, Stellenbosch University, Stellenbosch, Western Cape, South Africa

**Keywords:** autotrophic nitrification, comammox *Nitrospira*, pin-point flocs, tightly bound/ loosely bound EPS, ammonia removal, microbial community dynamics

## Abstract

The characteristics of biomass and microbial community dynamics, in relation to autotrophic nitrification, were studied in two 20 L stirred tank reactors (STR) with oxic/hypoxic/oxic zones. The bioreactors were fed with synthetic wastewater with stepwise increasing ammonia concentrations (50–200 N mg/L) without organic substrate in the first phase (autotrophic phase) for 35 days (R1) and 15 days (R2), followed by a heterotrophic phase (with supplementation of organic substrate). The settling properties of the biomass, represented by pin-point flocs, gradually improved in both reactors during the autotrophic phase. The pin-point flocs of R1 exhibited granule-like settling properties. The SVI_30_ in RI gradually improved to 29 mL/g MLSS, and the corresponding SVI_30_/ SVI_10_ gradually improved to 0.88 during the autotrophic phase. The settling properties of the biomass deteriorated in both bioreactors during the heterotrophic phase. The protein to polysaccharide ratio (PN:PS ratio) gradually increased in the extracted EPS (in both, loosely bound (LB) and tightly bound (TB) EPS) during the autotrophic phase, in both bioreactors. The TB:LB EPS ratio was higher when the pin-point flocs of R1 showed granule-like settling properties, followed by a decline in TB:LB EPS ratio during the heterotrophic phase. A combination of molecular approaches (droplet digital-PCR (dd-PCR) and 16S rRNA gene sequencing) revealed that *Nitrospira* were the predominant nitrifying bacteria in the pin-point flocs that show granular sludge-like settling properties during autotrophic phase in R1. Comammox *Nitrospira* was the dominant ammonia oxidizer in seed biomass and at low ammonia concentrations in both bioreactors. The relative abundance of canonical ammonia-oxidizing bacteria increased with an increase in influent-ammonia concentrations.

## Introduction

Ammonia, produced as a by-product in various domestic and industrial sources, such as agricultural and commercial processes, when released into the environment can lead to effects on human and ecosystem health (Soler et al., 2021). The ineffective removal of ammonia in wastewater treatment can result in eutrophication, as a result of increasing populations, and subsequently reducing oxygen levels that harm aquatic ecosystems (Ronan et al., 2021).

Conventional biological ammonia removal is a two-step process, i.e., nitrification and denitrification. Nitrification is a bottleneck in ammonia removal processes because nitrifiers compete with the heterotrophs for space and nutrients in the outer layers of biofilms and flocs. Autotrophic nitrifiers are slow-growing and are more prone to washout (Aqeel et al., 2019; Ronan et al., 2021; Christiaens et al., 2022). It has been reported that the proportion of nitrifying bacteria in wastewater treatment systems is as low as 0.4%, which is below the detection limit of certain molecular techniques such as dot blot and DNA hybridization (Dionisi et al., 2002). The nitrifiers are sensitive to fluctuations in operation conditions such as pH, temperature, and influent concentrations (Dionisi et al., 2002; Aqeel and Liss, 2020). In contrast, denitrifying bacteria are relatively more abundant in wastewater treatment systems because most denitrifying bacteria can use dissolved oxygen as an electron acceptor in the absence of nitrates (Aqeel et al., 2021; Christiaens et al., 2022).

Microbial aggregation, an important process in biological treatment, is mediated by extracellular polymeric substance (EPS) that holds the cells together (Flemming et al., 2023). The role of EPS is complex in determining the structural characteristics of the suspended biomass. The quantity of EPS is considered critical where too little or too much can result in poor settling or bulking sludge (Huang et al., 2022). Protein and polysaccharides are the major components of the EPS. The polysaccharides are largely considered the polymers that hydrate the flocs that help in the diffusion of nutrients. However, too much polysaccharide content results in high water retention and poor separation of treated water from the biomass. The protein content of EPS helps in the cohesion of microbial cells to form large and compact flocs or granules. Too little protein content can contribute to the formation of small pin-point flocs that result in non-filamentous bulking and system failure (Basuvaraj et al., 2015; Flemming et al., 2023). Several studies show that the ratio of protein to polysaccharide or the balance between protein and polysaccharides is vital in the formation of compact microbial aggregates and the settling efficiency of the flocs and granules (Liao et al., 2001; Aqeel et al., 2016; Aqeel and Liss, 2022). However, granular sludge with good settling properties has been reported with relatively higher polysaccharide content compared to protein in the EPS (Aqeel et al., 2019). There are a growing number of studies that indicate the two-layered structure of the flocs and granules. The outer layer is made up of loosely bound EPS and the core is made up of tightly bound EPS (Basuvaraj et al., 2015; Aqeel et al., 2019; Huang et al., 2022). The loosely bound EPS is rich in polysaccharide and loosely bound cells; whereas the inner layer of flocs and granules with tightly bound EPS is rich in protein content that forms a cohesive and dense core (Basuvaraj et al., 2015). The loosely bound EPS (rather than tightly bound EPS) dictates the settling properties of a floc or a granule (Huang et al., 2022). The loosely bound EPS is readily lost during the washing steps in most EPS extraction protocols (Basuvaraj et al., 2015). Therefore, contradicting reports about the composition of EPS and settling properties of the suspended biomass are present in the literature.

There is a need to optimize operational conditions to support the growth and prevent the washout of nitrifying bacteria from wastewater treatment systems. Overall, this study investigated the impact of autotrophic conditions and stepwise increase in influent ammonia concentration on the biomass characteristics and microbial community dynamics under oxic/hypoxic/oxic zones in STRs. The microbial community dynamics were characterized using 16S rRNA gene sequencing and digital droplet-PCR (dd-PCR). The dynamics of nitrifying bacteria (ammonia-oxidizing bacteria, *Nitrospira, and* comammox *Nitrospira*) with change in feeding regime, and the impact of the organic substrate was explored. The paper describes unique features on both the physical–chemical features of the microbial structures and the dominant nitrifying bacteria in the system studied.

## Materials and methods

### Experimental setup

Bioreactors 1 (R1) and 2 (R2) were operated continuously for more than 50 days. The 20 L working volume bioreactor was designed to support an initial oxic zone followed by a hypoxic zone (mixed but not aerated), and a final oxic zone. The dissolved oxygen concentration in the oxic/hypoxic/oxic chambers was 5–6 mg/L, <1 mg/L, and 5–6 mg/L, respectively. The hypoxic chamber was designed to support the growth of denitrifying bacteria, where endogenous decay would be a source of an organic substrate. Air pumps connected with stone air diffusers were added into both oxic chambers for aeration and mixing of the biomass. An electric overhead stirrer mixer was added into the middle hypoxic chamber. Peristaltic pumps were used for continuous feeding and return activated sludge (RAS). Draining was controlled passively from the settling tank.

R1 and R2 were seeded with RAS collected from the Ashbridges Bay municipal wastewater treatment plant (Toronto, Ontario, Canada). The seed biomass was collected twice (about two months apart) for R1 and R2, respectively. The seed biomass was largely composed of pin-point flocs with poor settling properties. The initial sludge volume index (SVI) of activated sludge of seed biomass for both reactors was 160–170 mL/g MLSS. The hydraulic retention time (HRT) of 16 h was maintained in the reactors. The solid retention time (SRT) was maintained at 12 to 15 days in both bioreactors. However, SRT in R1 declined (5–6 days) during phase-one when biomass washout was observed in the bioreactor. The proportion of oxic/hypoxic/oxic chambers were 40, 30, and 30% of total bioreactor, respectively. Therefore, HRT in the oxic/hypoxic/oxic chambers was six hours and 24 min, four hours and 48 min, and four hours and 48 min, respectively.

The bioreactors were fed with synthetic wastewater with stepwise increasing concentration of ammonia (50–200 N mg/L) without organic substrate during the autotrophic phase for 35 days (R1) and 15 days (R2). The autotrophic phases were maintained to support the enrichment of autotrophic nitrifying. The length of phase-one was different in both reactors to study its effect on characteristics of biomass and microbial community during autotrophic phase and subsequent heterotrophic phase. The synthetic feed used has been described previously (Aqeel and Liss, 2020). The autotrophic feed (without organic substrate) was composed of increasing concentrations of ammonium sulfate (50–200 N mg/L), sodium bicarbonate (354.32 mg/L), magnesium sulfate heptahydrate (70.98 mg/L), calcium chloride dihydrate (29.34 mg/L), potassium dihydrogen phosphate (79.09 mg/L), iron (II) sulfate heptahydrate (4.98 mg/L), and the micronutrients.

### EPS extraction

Biomass samples for EPS extraction were collected from the final oxic zone. Loosely bound (LB) and tightly bound (TB) EPS were extracted following a previous study (Basuvaraj et al., 2015). Briefly, biomass samples (35 mL) were collected from the bioreactors and centrifuged in a 50 mL falcon tube at 3000 g for 5 min. The supernatant was decanted, and the pellet was processed for LB and TB EPS extraction. The pellet was resuspended in the EPS extraction buffer (0.002 M Na_3_PO_4_, 0.004 mM NaH_2_PO_4_, 0.009 mM NaCl and 0.001 mM KCl) and vortexed for 1 min, followed by centrifugation at 4000 g for 10 min. The supernatant (LB EPS) was stored in a − 80°C freezer. The pellet was resuspended in the EPS extraction buffer and pre-washed cation exchange resin (CER) (DowexR, Na + form, Sigma Aldrich). The resuspended pellet was vortexed for 60 min. Afterward, tubes were centrifuged at 9000 g for 10 min, and the supernatant (extracted TB EPS) was stored in a − 80°C freezer (Basuvaraj et al., 2015). The protein and polysaccharide content in the extracted EPS was quantified as described previously by Aqeel et al. (2016). The EPS samples were stored in a − 80°C freezer and all samples were processed together for protein and polysaccharide quantification to minimize the bias.

### Microbial community composition

#### DNA extraction

Biomass samples were collected and stored in a − 80°C freezer. Nucleic acid from all biomass samples was extracted using a QIAGEN QIAcube Connect (Qiagen, MD, USA) to automate the DNA extraction procedure. Approximately 100–150 mg of the wet pellet was used for DNA extraction. In the final step of the DNA extraction, 100 μL of nucleic acid-free water was used to elute the nucleic acid from the spin column. The DNA concentration was measured using a NanoPhotometer™ Pearl (Implen, Münchem, Germany). The DNA was then stored in a − 80°C freezer until downstream analysis.

#### Digital droplet-PCR analysis

Quantification of total bacteria (EUB) and nitrifying bacteria (ammonia-oxidizing bacteria (AmoA), *Nitrobacter* (Nitro), *Nitrospira* (NSR), and comammox) was performed using digital droplet-PCR (dd-PCR). For the detection of comammox *Nitrospira*, two sets of primers (for clade A and B) were used: comaA-244F and coma-659 R and comaB-244F and comaB-659R (Pjevac et al., 2017). The primer sequences used to target EUB, AmoA, *Nitrobacter*, *Nitrospira* and Comammox are presented in Table 1. The primers were purchased from IDT (IDT, Coralville, Iowa, USA). The PCR conditions for each primer set were optimized by the thermal gradient cycles and template dilutions. The 10-fold dilutions of each sample were prepared in nuclease-free water. Based on the template concentration optimization results, three 10-fold serial dilutions were used as replicates for the dd-PCR analyses. The PCR plates were sealed using PX1™ PCR plate sealer (BioRad), and vortexed for one minute. The PCR plate was then centrifuged for 40 s in a mini plate centrifuge (VWR, Canada). The droplets were generated using an automated droplet generator (BioRad). The PCR reaction was performed in a thermal cycler (C1000 Touch™ Thermal cycler, BioRad). The droplets were read using QX200™ Droplet Reader (BioRad). The dd-PCR results were analyzed using QX manager (QX200™, BioRad).

**Table 1:** Primer targets and sequences used for quantification microbial population by dd-PCR.

Primer pair	Target	Forward (5′-3′)	Reverse (5′-3′)	Reference
341f-518R	EUB	CCTACGGGAGGCAGCAG	ATTACCGCGGCTGCTGG	Agrawal et al. (2021)
AmoA1f-amoA2r	AOB	GGGGTTTCTACTGGTGGT	CCCCTCKGSAAAGCCTTCTTC	Agrawal et al. (2021)
Nitro1198f-Nitro1423r	*Nitrobacter*	CGGTTTTTTGAGATTTGCTAGGGGT	CTTCACCCCAGTCGCTGACC	Agrawal et al. (2021)
Nspra675f-Nspra-746r	*Nitrospira*	GCGGTGAAATGCGTAGAKATCG	TCAGCGTCAGRWAYGTTCCAGAG	Agrawal et al. (2021)
ComaA-244F- comaA-659R	Comammox clade A	TAYAAYTGGGTSAAYTA	ARATCATSGTGCTRTG	Pjevac et al. (2017)
ComaB-244F- comaB-659R0	Comammox clade B	TAYTTCTGGACRTIYTA	ARATCCARACDGTGTG	Pjevac et al. (2017)

#### 16S rRNA gene sequencing

The genomic DNA samples were sent to the Genome Quebec Research and Testing Laboratory in Montréal, Québec for 16S rRNA gene sequencing (Illumina MiSeq). The DNA samples were amplified using PCR for amplicon preparations. The V3–V4 region of the 16S rRNA gene was amplified using primers 341F (CCTACGGGNGGCWGCAG) and 803R (CTACCRGGGT ATCTAATCC). A second PCR reaction was performed to incorporate sample-specific barcodes. The DNA concentration of all PCR reactions was measured using Picogreen so that the equimolar concentration of all samples could be used for sequencing. The amplicon library with an insert size of about 450 bases was sequenced with a paired-end 250 kit (Illumina MiSeq). The forward and reverse sequences from the Illumina paired-end sequences were merged using the DADA2 software. The markers, adapters, and errors in the sequences (including chimera sequences) were removed using the DADA2 pipeline (Callahan et al., 2016), 16S rRNA gene sequences were used to generate an operational taxonomic unit (OTU) table and a corresponding FASTA file. The sequences after the removal of chimeras and low-quality sequences, were analyzed using Microbiome Analyst. The assignment of the sequences was performed using SILVA taxonomy (Dhariwal et al., 2017; Chong et al., 2020) for alpha diversity analyses (based on observed species), and for relative abundance of bacteria. The 16S rRNA gene sequencing data have been submitted to the National Library of Medicine, National Center for Biotechnology Information. The sequence read archive (SRA) submission number is SUB13878507, and BioProject number is PRJNA1023363.

## Results

### Bioreactor performance and biomass characteristics

**Figure 1:**
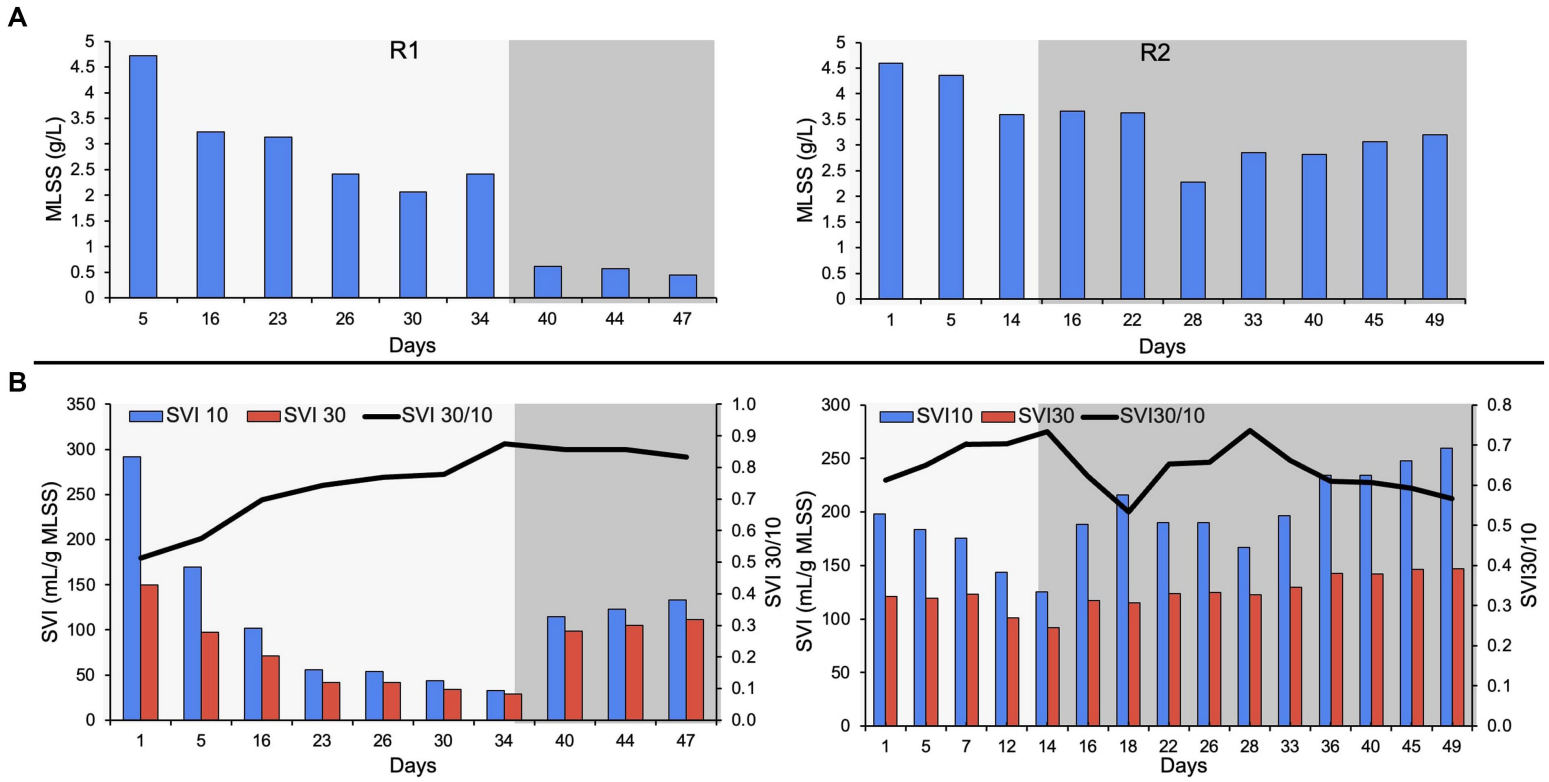
**(A)** MLSS in R1 and R2, **(B)** settleability of the biomass showing the SVI_30_, SVI_10_ and SVI_30_/ SVI_10_. The lighter shade indicates phase one and the darker shade indicates phase two.

The bioreactors were seeded with RAS from the Ashbridges Bay municipal wastewater treatment plant (Toronto, Ontario, Canada). The MLSS in R2 was relatively lower (4.4 g/L) compared to R1 (4.7 g/L), on day five of bioreactor startup (Figure 1). The biomass washout is expected during reactor startup due to a change in influent feed, from municipal wastewater to autotrophic synthetic feed. The ESS in both reactors was stabilized to below 100 mg/L after day 15 of bioreactor startup. The R2 was switched to phase-two on day 15 (the bioreactor with a short autotrophic phase). A transient increase in ESS was observed during the switch from phase-one to phase-two, in both reactors. The MLSS in R1 gradually declined to 2.07 g/L on day 30, during phase-one. The biomass loss due to the switch in feed (phase-two on day 35) resulted in very low MLSS (0.61 g/L) on day 40. The switch in feeding regime resulted in poorly settling and bulking sludge that resulted in biomass washout in R1. However, the bioreactor with a short autotrophic enrichment phase (R2) recovered after the switch in the feeding regime during phase-two. The MLSS in R2 was relatively higher (3.2 g/L) compared to R1 (0.45 g/ L), at the end of the experiment (Figure 1).

Dense pin-point flocs 10–20 μm in size were observed during the autotrophic phase (phase-one) in both bioreactors (Supplementary Figure S1). During the autotrophic phase, the settling properties of the biomass gradually improved in both bioreactors with the formation of compact pin-point flocs. The SVI_30_ (ml/g MLSS), SVI_10_ (ml/g MLSS), and the ratio of SVI_30_ to SVI_10_ (SVI_30_/ SVI_10_) were used to characterize the settling properties of the flocs (Figure 1). SVI_30_ improved to 75 and 92 in R1 and R2 respectively, on day 15 of bioreactor operation. The SVI_30_/ SVI_10_ of both bioreactors was 0.7 after 15 days of bioreactor operation during the autotrophic phase (Figure 1). In R1, the SVI_30_ improved to 29 mL/g MLSS, and the SVI_30_/ SVI_10_ gradually improved to 0.88 on day 34 (Figure 1). However, after a relatively longer autotrophic phase, when the organic substrate was supplemented to R1 (during phase-two), the flocs were showing poor settling properties and biomass washout. The SVI_30_ declined from 29 to 111 mL/g MLSS and MLSS declined from 2.42 to 0.45 g/L, with the switch in synthetic feed composition. In R2, the SVI_30_ gradually increased to 147 mL/g MLSS, on day 49 of reactor operation. Whereas, in R2, the SVI_30_/ SVI_10_ stabilized at 0.6 ± 0.1, and MLSS was maintained above 3 g/L (Figure 1). The settling properties of the biomass deteriorated in both reactors, after the supplementation of organic substrate (during phase-two).

**Figure 2:**
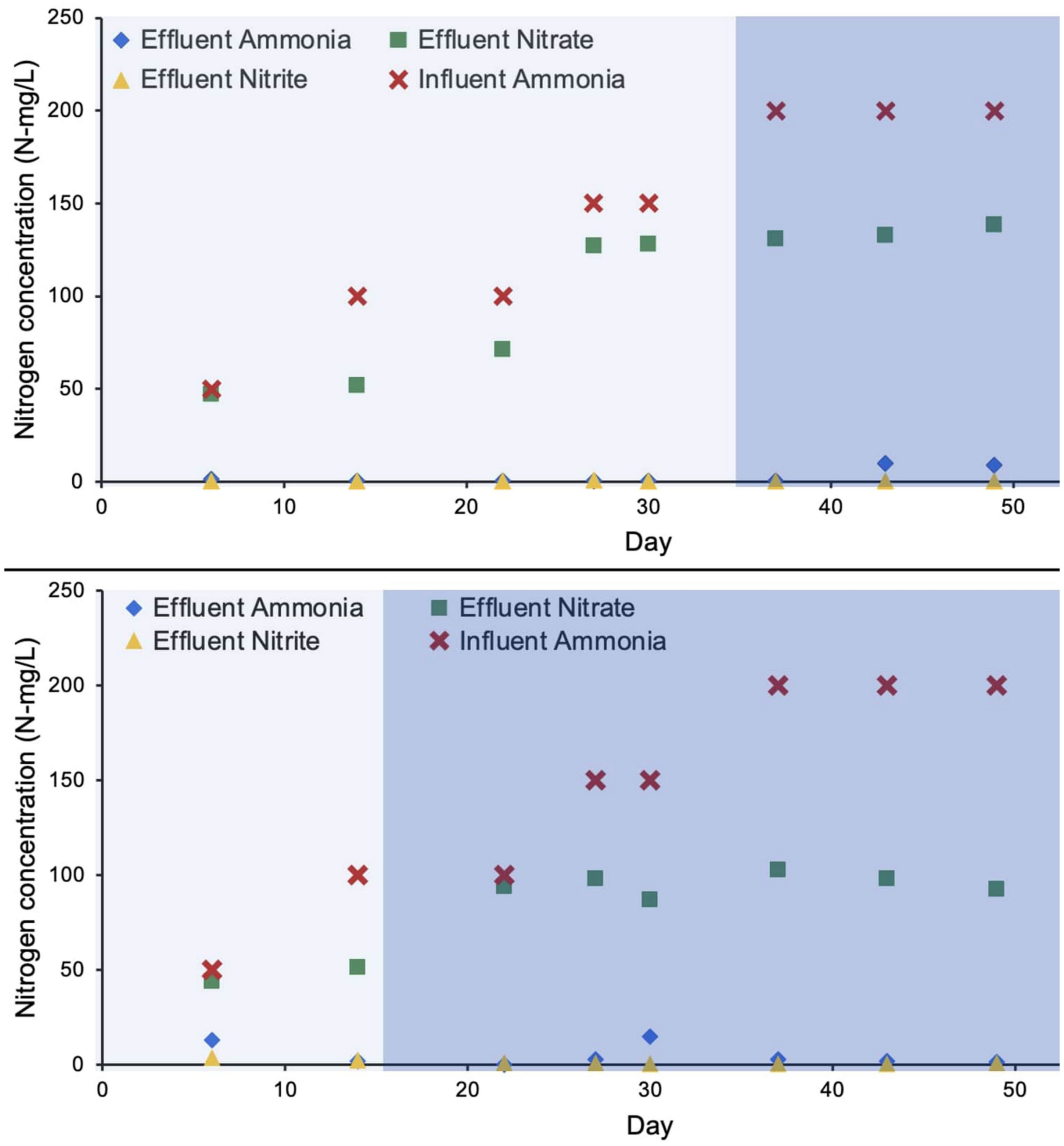
Concentration of nitrogen species during the bioreactor operation. The lighter shade indicates phase one and the darker shade indicates phase two.

Ammonia removal efficiency for R1 and R2 over the duration of approximately 50 days was 98.5 and 96.6%, respectively. The nitrification efficiency was 100% during the autotrophic phase, however, a drop in ammonia removal was observed during the heterotrophic phase (Figure 2). R1 and R2 maintained their nearly complete ammonia removal efficiency as higher concentrations of influent ammonia increased to 200 N mg/L (Figure 2). Nitrite concentrations in both reactors were also limited to nearly zero, as the average in each bioreactor was 0.15 mg/L and 1.27 mg/L, respectively. The nitrate accumulation was relatively higher in R1 to a maximum concentration of 132 N mg/L on day 49 of bioreactor startup. In R2 (the bioreactor operated with a shorter autotrophic phase) the nitrate accumulation was relatively lower (98 N mg/L) on day 49 (Figure 2).

### Composition of extracellular matrix

**Figure 3:**
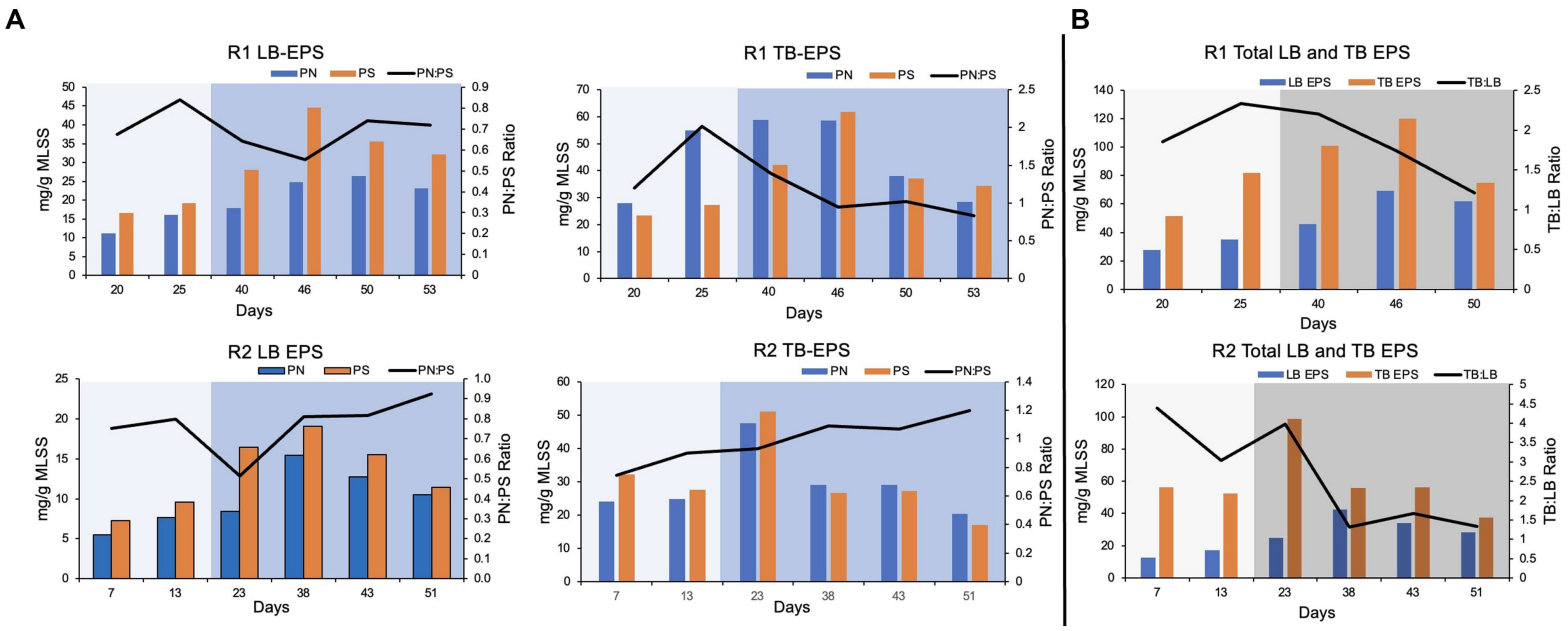
EPS composition. **(A)** Protein (PN) and polysaccharide (PS) content and PN:PS in the loosely (LB) and tightly bound (TB) extracted EPS. **(B)** TB and LB EPS and TB:LB ratio in the extracted EPS. The lighter shade indicates phase one and the darker shade indicates phase two.

In this study, loosely bound (LB) and tightly bound (TB) EPS were extracted separately from the biomass. The protein and polysaccharide content in each fraction of EPS was quantified. Overall, the polysaccharide concentration was relatively higher compared to the protein content, in LB EPS. It was reflected by a low protein to polysaccharide ratio (PN:PS ratio) in LB EPS extracted from pin-point flocs of R1 and R2 (Figure 3). The protein concentration was relatively higher (up to four-fold) in the TB EPS fractions compared to the respective LB EPS fractions (Figure 3).

In R1, protein and polysaccharide concentration in LB and TB EPS gradually increased during phase-one (Figure 3). The PN:PS ratio gradually decreased in both LB and TB EPS during phase-two, in R1. The PN:PS ratio in the TB EPS declined from 2.02 (during phase-one on day 25) to 0.95 (on day 46, during phase-two). In R2, a similar trend was observed during the autotrophic phase where an increase in PN:PS ratio was observed in both LB and TB EPS. However, in R2 (the bioreactor with a shorter phase-one) the PN:PS kept on gradually improving in TB EPS, during phase-two (Figure 3). In R1 the ratio of TB to LB EPS increased during the autotrophic phase followed by a decline in TB:LB ratio during the heterotrophic phase (Figure 3) when the organic substrate was supplemented with the synthetic feed. A similar trend was observed in R2, where TB:LB ratio gradually declined during phase-two (Figure 3).

### Microbial community composition

#### 16S rRNA gene sequencing

**Figure 4:**
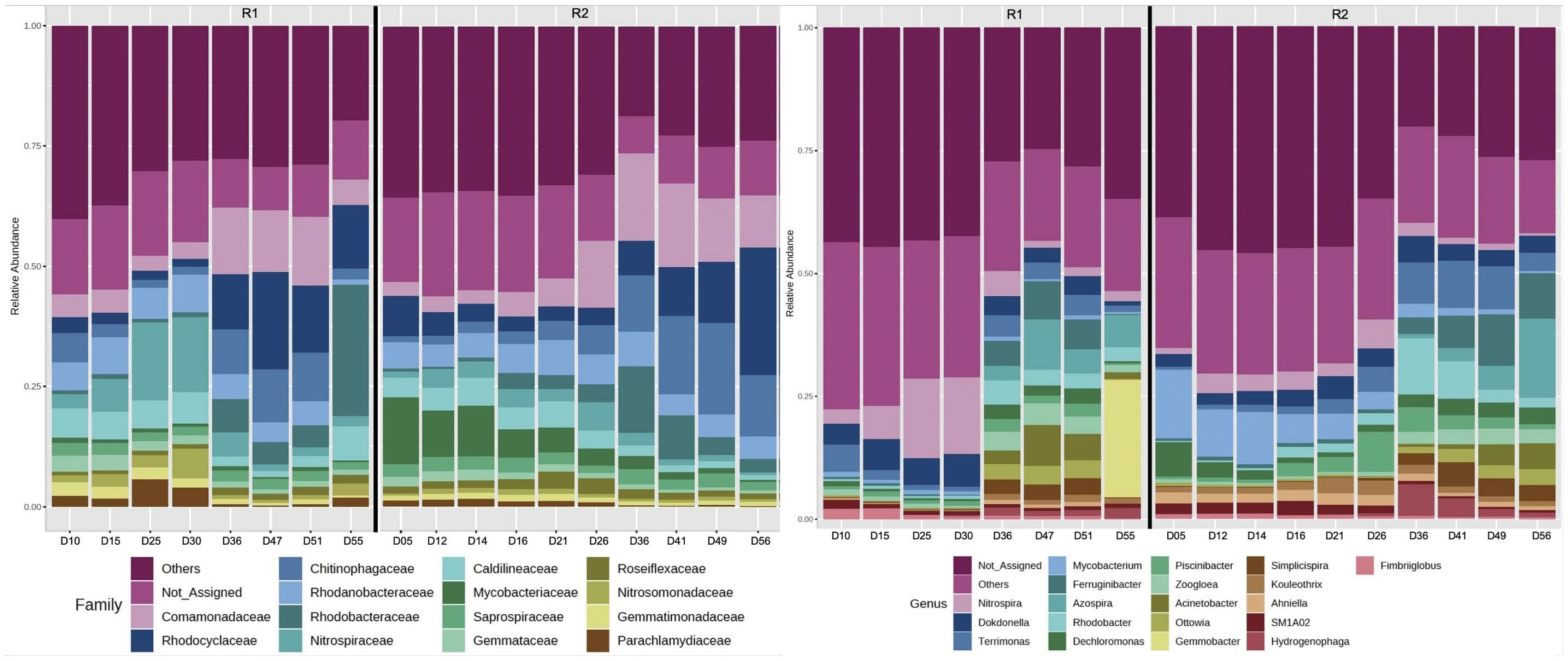
16S rRNA gene sequencing (Illumina MiSeq) showing the relative abundance of the most abundant families, and the relative abundance of the 20 most abundant genera. Phase one was up to day 35 in R1 and day 15 in R2.

The 16S rRNA gene sequencing data (Illumina MiSeq PE 250) was analyzed using the Microbiome Analyst. Overall, the alpha diversity analyses (based on observed species) revealed that the microbial communities of R2 were relatively more diverse compared to R1 (Supplementary Figure S1). The relative abundance of bacteria related to families *Comamonadaceae* and *Rhodocyclaceae* gradually declined during the autotrophic enrichment phase in both bioreactors. The relative abundance of *Comamonadaceae* declined from 10.8 to 3.6% on day 34, followed by an increase in relative abundance to 14.5% on day 51 in R1 (Figure 4). In R2, the relative abundance of *Comamonadaceae* increased from 2.9% (on day 14 of bioreactor operation) to 13.3% (on day 49 of R2 operation). Similarly, the relative abundance of *Rhodocyclaceae* gradually declined from 9 to 1.5% on day 34, followed by an increase in relative abundance to 20% (on day 47 of R1 startup). In R2, the relative abundance of *Rhodocyclaceae* gradually declined from 10.5 to 3.7% during phase-one. During phase-two, the relative abundance of *Rhodocyclaceae* gradually increased to 26.4% (on day 56 of R2 startup). The relative abundance of bacteria related to the family *Nitrospiraceae* was higher during the autotrophic phase and then declined during the second phase when the organic substrate was supplemented into the synthetic feed. The relative abundance of *Nitrospiraceae* gradually increased from 0.1 to 15.4% (on day 34 of R1 operation) during phase-one (Figure 4). The relative abundance of *Nitrospiraceae* declined to 1.8% (on day 51 of R1 operation) during phase-two. In R2, the relative abundance of *Nitrospiraceae* gradually increased from 0.6 to 3.4% on day 14 of bioreactor operation. During phase-two, the relative abundance of *Nitrospiraceae* gradually decreased to 0.5% (on day 56 of R2 startup).

At the genus level, it was observed that the relative abundance of *Nitrospira*, *Dokdonella*, and *Candidatus Accumulibacter* was higher in both bioreactors during phase-one compared to phase-two of the feeding regime (Figure 4). The relative abundance of *Thauera*, *Zoogloea*, *Acinetobacter*, *Comamonas*, *Rhodobacter*, and *Dechloromonas* was relatively higher during phase-two (when the organic substrate was supplemented into the synthetic feed). At the species level, most of the low-count OTUs were filtered out, and only very few bacteria species were identified. As a result, analyses at the species level were not undertaken. However, the observation of a comammox species based on 16S rRNA gene sequencing warranted the quantification of comammox bacteria using dd-PCR.

#### Digital droplet-PCR

**Figure 5:**
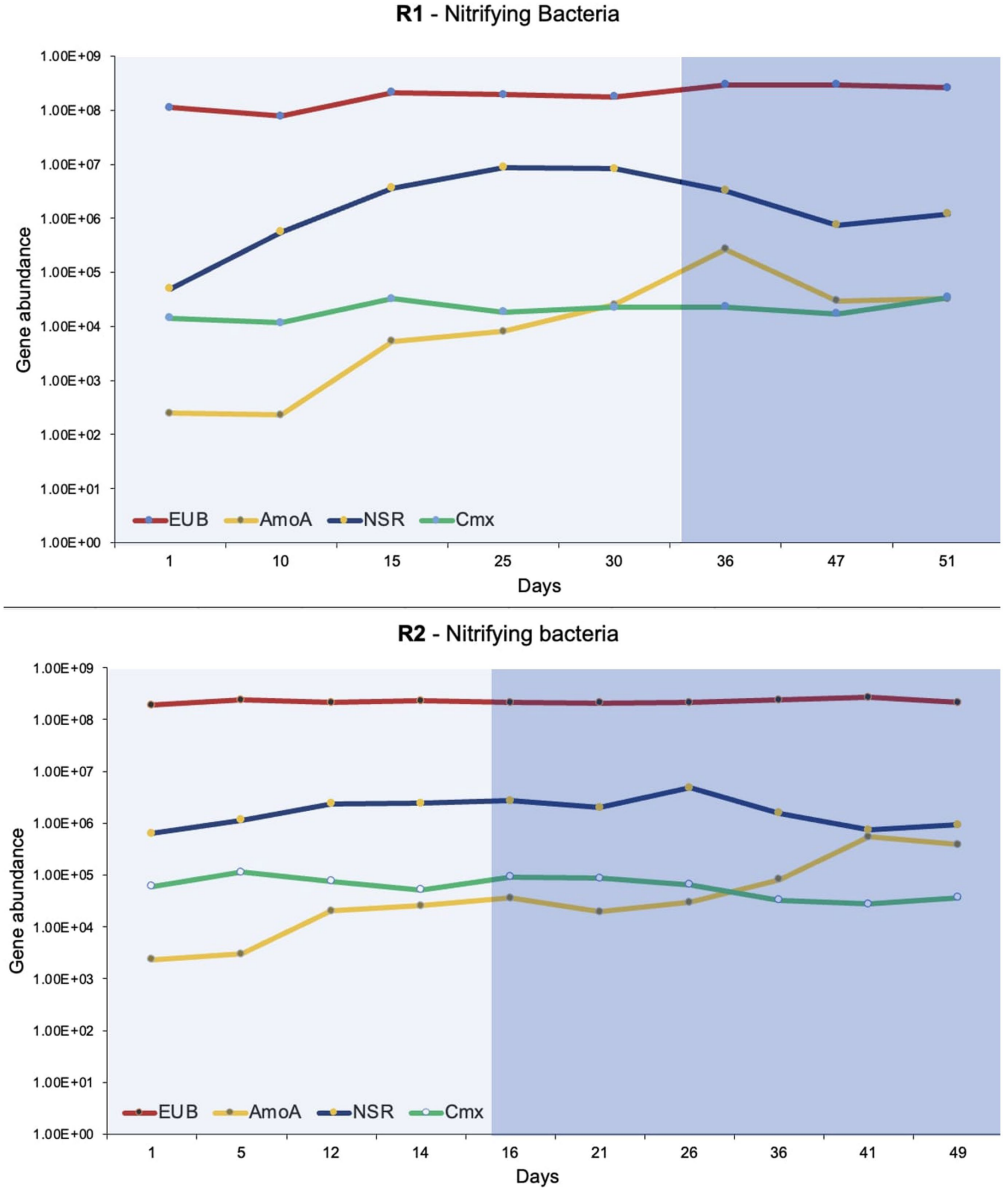
The dd-PCR results show the abundance of total bacteria (EUB), ammonia-oxidizing bacteria (AmoA), *Nitrospira* (NSR), and comammox (Cmx). The lighter shade indicates phase one and the darker shade indicates phase two.

The dd-PCR was used to quantify total bacteria (EUB), *Nitrospira*, *Nitrobacter*, comammox, and ammonia-oxidizing bacteria (Figure 5). The primer sequences and targets used for quantification of bacteria are presented in Table 1. The dd-PCR has an upper limit of detection that can read up to 10^4^ to 10^5^ gene copies (depending on the amount of DNA templates used). Two or three successive serial dilutions were used for duplicate or triplicate dd-PCR experiments. It was helpful to detect the concentration of a bacteria that fluctuates with bioreactor operation. Additionally, the dd-PCR on two or three serial dilutions confirms the accuracy of the manual serial dilution of the samples. Total bacteria ranged from 10^7^ to 10^8^ gene copies/ ug of DNA extracted in both bioreactors. *Nitrobacter* was not detected (or amplified) in the DNA extracted from the flocs of both bioreactors. The enrichment of the *Nitrospira* was observed in both bioreactors during phase-one when the synthetic feed lacked organic substrate. *Nitrospira* were the predominant nitrifying bacteria in both bioreactors and ranged from 10^4^ to 10^6^ gene copies/ ug of DNA extracted. The highest number of *Nitrospira* was observed when pin-point flocs showed granule-like settling properties (Figure 5). The abundance of comammox *Nitrospira* in the biomass samples was measured using dd-PCR when *Nitrospira Nitrosa* (a comammox *Nitrospira*) was observed in the 16S rRNA gene sequencing results. Overall, the abundance of comammox *Nitrospira* remained relatively constant in both bioreactors. The dd-PCR results show that the comammox *Nitrospira* were the predominant ammonia-oxidizing bacteria in the seed biomass collected from the municipal wastewater treatment plant and at low ammonia influent concentrations in the bioreactors. The abundance of ammonia-oxidizing bacteria increased from 10^2^ to 10^5^ with a stepwise increase in influent ammonia concentration (Figure 5).

## Discussion

The characteristics of the biomass and microbial community dynamics were studied during autotrophic and heterotrophic phases in the STRs operated under oxic/hypoxic/oxic zones. The R1 was operated with a relatively prolonged autotrophic phase (phase-one), compared to R2. The organic substrate was supplemented in phase-two to support the growth of heterotrophic denitrifying bacteria. Pin-point flocs with granular-sludge-like settling properties were formed in a 20 L stirred tank reactor (STR) operated with a relatively longer autotrophic phase (Supplementary Figure S1). The settling properties of the pin-point flocs improved gradually during the autotrophic enrichment phase. Whereas in the second phase with the supplementation of the organic substrate, the settling properties of biomass deteriorated in both bioreactors. The study shows that autotrophic bacteria improved the settling performance and heterotrophic bacteria deteriorated the settleability of the biomass.

Additionally, the operating conditions for this study were planned to understand the microbial community dynamics (of the nitrifying bacteria) with stepwise increasing influent ammonia concentrations. The stepwise increment in the influent ammonia concentration in synthetic feed (without organic substrate) has been used previously for the enrichment of autotrophic nitrifying bacteria (Aqeel and Liss, 2020). Microbial community dynamics of the k-strategist and r-strategist nitrifying bacteria were studied in the bioreactors operated with oxic/hypoxic/oxic zones. K-strategists are slow-growing bacteria (such as *Nitrospira*) with a high affinity for a substrate. R-strategists are fast-growing bacteria (such as *Nitrobacter*) with low affinity for a substrate (Yin et al., 2022). Filamentous bacteria are also k-strategist microbes because they are better adapted to limited resources due to their relatively higher surface area (Yin et al., 2022). To inhibit the growth of heterotrophic filamentous bacteria, the synthetic feed was made without an organic substrate. Furthermore, a hypoxic chamber was designed to support the growth of heterotrophic denitrifying bacteria that can use organic substrate from the endogenous decay, and oxidized nitrogen species as electron acceptors (Aqeel et al., 2019). Previous studies have shown that low DO concentrations inhibit filamentous growth (Adav et al., 2007; Aqeel et al., 2021). It was observed that in the present study, the filamentous bacteria that were present in the seed biomass were inhibited in both bioreactors, during the autotrophic phase.

### Granule-like settling properties of pin-point flocs

The pin-point flocs are generally correlated to the poor settling of activated sludge. The seed biomass was predominantly composed of pin-point flocs that show poor settling properties (SVI of 160–170 mL/g MLSS). Enrichment of compact pin-point flocs and inhibition of filamentous bacteria was observed during the autotrophic phase that showed granule-like settling properties, in R1. The SVI_30_ and ratio of SVI_30_/ SVI_10_ are used to show the quality and completeness of granular sludge. The SVI_30_ of granular sludge is usually less than 50 mL/g MLSS (Derlon et al., 2016; Sarma and Tay, 2018). In this study, the SVI_30_ improved to 29 mL/g MLSS during the autotrophic enrichment phase in R1 (Figure 1). The SVI_30_/ SVI_10_ of close to 1 indicates the completeness of granular sludge (Schwarzenbeck et al., 2005; Aqeel and Liss, 2022). However, most granular sludge studies report ratios of SVI_30_/ SVI_10_ between 0.7 to 0.9 (Derlon et al., 2016; Manavi et al., 2017; Aqeel et al., 2021). In this study, the SVI_30/10_ of pin-point flocs improved to 0.88 on day 34 during the autotrophic enrichment phase (Figure 1).

The settling properties of R1 and R2 biomass were similar, during the autotrophic phase on day 15 (SVI_30_/ SVI_10_ was 0.7 in both bioreactors). The R1 biomass showed granule-like settling properties on day 25. However, the autotrophic enrichment phase was relatively shorter in R2 (15 days). Therefore, the settling properties of the R2 biomass improved, but it was not like the settleability of a granular sludge. The settling properties of the biomass deteriorated during phase-two, in both bioreactors (Figure 1). The settling properties of R2 biomass did not improve like the settling properties of R1 biomass (during the autotrophic phase). However, the settling properties of R2 biomass did not deteriorate drastically, like R1 biomass, during the switch in the feeding regime.

The alpha diversity analyses of the microbial communities in R1 and R2 revealed that the microbial diversity was relatively lower in R1 which was operated with longer phase-one (Supplementary Figure S2). It was observed that the microbial selection/ enrichment during phase-one resulted in the enrichment of nitrifying bacteria (Figures 4, 5) and compact pin-point flocs that show granule-like settling properties (Figure 1). However, it resulted in the loss of the resilience of the bioreactor to changes in nutritional conditions. Therefore, during phase-two, sludge bulking was observed in R1 with an abundance of *Zoogloea* in the microbial community (Figure 4). The correlation between lack of microbial diversity during granule formation and subsequent granule instability has also been observed in granular sludge technology. Therefore, optimization of granule enrichment and the hybrid granulation (balance between flocs and granules) have been suggested for the stability of granular sludge (Aqeel et al., 2019). Similarly in this study, it was observed that where autotrophic conditions can propagate the enrichment of nitrifying bacteria and improve the settling properties of the biomass; optimization of conditions to find a balance is important for the stable operation of the biological nutrient removal bioreactors.

### Extracellular matrix

The extracted loosely bound (LB) and tightly bound (TB) EPS content and composition are presented in Figure 3. The protein to polysaccharide ratio (PN:PS ratio) increased during the autotrophic phase in the TB and LB EPS extracted from pin-point flocs of both bioreactors. Basuvaraj et al. (2015) characterized the granular sludge and flocs, based on PN:PS ratio. The PN:PS ratio of the granular sludge was higher than 1.5 and the PN:PS ratio of the flocs was below one (Basuvaraj et al., 2015). In R1, the highest PN:PS ratio (>2) was observed on day 25 of bioreactor startup, when pin-point flocs were observed and exhibited granule-like settling properties. The PN:PS ratio in TB EPS extracted from R1, gradually decreased during phase-two which corresponds to the poor settling properties of the pin-point flocs (Figure 3). The PN:PS ratio in TB EPS extracted from R2 gradually increased with the bioreactor operation that corresponds to the stability in the settling properties of the R2 pin-point flocs during phase-two (Figure 1). The autotrophic enrichment phase resulted in the selection of compact flocs that show settling properties and EPS composition of a granular sludge, in R1. However, the PN:PS ratio and settling properties of the biomass during phase-two indicate that the R2 (that was operated with a shorter phase-one) better adapted to the change in feeding regime compared to the R1.

The TB and LB EPS fractions were extracted separately because the ratio of TB and LB EPS is an important indicator of the settling characteristics of the suspended biomass (Basuvaraj et al., 2015; Chen et al., 2019). The protein content in TB EPS was higher than the LB EPS, in both bioreactors. The PN:PS ratio was higher in TB EPS compared to the LB EPS in both bioreactors (Figure 3). The results are consistent with previous studies that show protein content in the extracellular matrix is associated with the formation of cohesive and compact flocs (Liao et al., 2001; Basuvaraj et al., 2015; Aqeel et al., 2019). Therefore, TB EPS is more associated with the cohesiveness of flocs and LB EPS is associated with poor settleability of the flocs (Bezawada et al., 2013; Basuvaraj et al., 2015; Huang et al., 2022). Basuvaraj et al. (2015) differentiated the granular sludge and flocs based on the ratio of TB and LB EPS. It was reported that the TB:LB ratio is higher in the granular sludge compared to the flocs (Basuvaraj et al., 2015). In R1, the TB:LB ratio gradually increased during the autotrophic phase (Figure 3) with the observation of pin-point flocs that show granular-sludge-like settling properties (Figure 1). The TB:LB ratio of the pin-point flocs in R1 during phase-two gradually declined that correspond with poor settling properties.

The nitrifiers form small but very strong microcolonies, therefore most EPS extraction methods can not completely extract EPS (Huang et al., 2022). Nitrifying microcolonies remain largely intact under extreme physical and chemical treatments. *Nitrosomon*as and *Nitrospira* microcolonies are formed by physical entanglement of the extracellular polymers. Where *Nitrospira* microcolonies are relatively more cohesive than the *Nitrosomonas* microcolonies (Daims et al., 2001; Larsen et al., 2008). The nitrifying bacteria were enriched in this study, during the autotrophic phase in both bioreactors. Therefore, it is expected that the TB EPS was not completely extracted from the biomass. Although the TB:LB EPS ratio reported in this study is similar to a granular sludge; the actual TB EPS might have been higher in the biomass.

Compact pin-point flocs up to 20 μm in size were predominant during the autotrophic phase in R1 and R2. Previous studies have shown that nitrifying bacteria form dense microcolonies in the ranges from 9-25 μm (Larsen et al., 2008), similar to the pin-point flocs observed in this study. It is hypothesized that these pin-point flocs consist predominantly of nitrifying microcolonies. The autotrophic growth conditions presented in this study inhibited the growth of heterotrophic bacteria including filamentous bacteria which improved the overall settling properties of the biomass. Both molecular approaches (16S rRNA gene sequencing, and dd-PCR) show that nitrifying bacteria were predominant during the autotrophic phase. The relative abundance of *Nitrospira* was highest when the pin-point flocs of R1 showed granule-like settling properties. The abundance of nitrifying bacteria translated into 100% ammonia and nitrite removal efficiencies during the autotrophic, in R1 (Figure 2). However, the decline in ammonia removal efficiencies during phase-two, could be due to biomass washout in R1 (Figure 1).

### Microbial community

16S rRNA gene sequencing revealed that the change in feeding regime resulted in a clear shift in microbial community from autotrophic nitrifying to heterotrophic denitrifying bacteria (Figure 4). For example, during phase-one autotrophic bacteria (*Nitrospira*) were predominant in the microbial community. Furthermore, the relative abundance of bacteria related to families *Rhodocyclaceae* and *Comamonadaceae* [known for denitrification (Pishgar et al., 2019)] gradually declined during the autotrophic phase in both bioreactors. During phase-two the heterotrophic bacteria were relatively more abundant. It was observed that at the family level the relative abundance of *Rhodocyclaceae* and *Comamonadaceae* increased, during phase-two in both bioreactors (Figure 4). Whereas the relative abundance of the dominant nitrifying bacteria (*Nitrospira*) declined during phase-two. At the genus level, it was observed that the aerobic denitrifier *Dokdonella* (Pishgar et al., 2019) was relatively a predominant denitrifier during phase-one in both bioreactors. During phase-two, *Acinetobacter*, *Simplicispira*, and *Dechloromonas* [denitrifiers (Pishgar et al., 2019)] were predominant in both bioreactors (Figure 4). *Dechloromonas* have also been found associated with denitrifying phosphorus accumulation organism (DPAO) (Weissbrodt et al., 2014). It is suggested that the oxic/hypoxic/oxic zones favored the growth of *Dechloromonas* in the bioreactors. The abundance of *Dechloromonas* has been observed previously in the laboratory-scale sequencing batch bioreactors operated with oxic/hypoxic/oxic conditions (Aqeel et al., 2021).

The abundance and microbial community dynamics of nitrifying bacteria were quantified using dd-PCR (Figure 5). It was observed that comammox *Nitrospira* was the dominant ammonia oxidizer in the seed biomass that was collected from a full-scale Ashbridges Bay wastewater treatment plant. Additionally, it was the dominant ammonia-oxidizer in both bioreactors at low ammonia influent concentrations (Figure 5). The comammox has relatively more affinity for dissolved oxygen and ammonia compared to the *Nitrosomonas* species. Therefore, comammox *Nitrospira* are capable to outcompete the ammonia-oxidizing bacteria under substrate-limiting conditions (Roots et al., 2019; Ren et al., 2020; Zhu et al., 2022). The comammox *Nitrospira* were largely identified in oligotrophic conditions such as drinking water treatment plants (Pinto et al., 2016; Maddela et al., 2022) or a biofilm growing 1,200 m below the surface of an oil exploration well (Daims et al., 2015; Roots et al., 2019). Therefore, it is considered that the canonical ammonia oxidizing bacteria may outcompete the comammox bacteria in nutrient-rich conditions in wastewater treatment plants (Roots et al., 2019). The hypothesis was verified by several initial studies that show comammox *Nitrospira* were absent or relatively low in abundance compared to ammonia-oxidizing bacteria in biological nutrient removal systems. Therefore, it was considered that comammox is irrelevant or less important in nitrogen cycling (Gonzalez-Martinez et al., 2016; Annavajhala et al., 2018). There is a growing number of evidence that shows an abundance of comammox *Nitrospira* in wastewater treatment systems, specifically at low dissolved oxygen concentrations (Roots et al., 2019).

Overall, the abundance of comammox *Nitrospira* remained constant during stepwise increments in influent ammonia concentration and during shifts in the feeding regime (phase-one and phase-two), in both bioreactors (Figure 5). The observation corresponds to the physiological and phylogenetic analyses that suggest comammox *Nitrospira* are resilient microorganisms that can withstand ammonia fluctuation and oxidative stress (Maddela et al., 2022).

The relative abundance of ammonia-oxidizing bacteria (compared to comammox *Nitrospira*) gradually increased with an increase in influent ammonia concentrations in both bioreactors (Figure 5). Many ammonia-oxidizing bacteria are r-strategists that are favored by increasing the concentration of the substrate (Yin et al., 2022). The results of the present study showed the dynamics of comammox *Nitrospira* and ammonia-oxidizing bacteria with increasing substrate concentrations. The insight will help to optimize the conditions to find a balance between the r and k strategist nitrifiers to treat high-strength ammonia wastewater and produce high-quality effluent.

The second step of nitrification is carried out by canonical nitrite-oxidizing bacteria (*Nitrospira* and *Nitrobacter*) and comammox bacteria. The two canonical nitrite-oxidizing bacteria compete for a common substrate where *Nitrobacter* is an r-strategist and *Nitrospira* is a k-strategist bacteria (Mehrani et al., 2023). The presence of *Nitrobacter* in a system is correlated to poor nitrification removal (Terada et al., 2010). The dd-PCR and 16SrRNA gene sequencing results show that *Nitrobacter* was absent (or below the detection limit) in both bioreactors. Both molecular approaches showed a gradual increase in *Nitrospira* abundance during phase-one (Figures 4, 5). The k-strategist nitrifying bacteria are known for polishing and producing high-quality effluent. In the present study up to 100% nitrification was observed during phase-one with the enrichment of *Nitrospira*.

In the present study, the stepwise incremental increase of ammonia concentration in the absence of organic substrate supported the enrichment of k-strategist bacteria, because there was minimal excess substrate for the growth of r-strategists. The abundance of k-strategists was correlated with the abundance of compact pin-point flocs and the absence of filamentous bacteria. The retention of pin-point flocs was observed to be correlated to the granule-like properties reflected in the EPS (TB/LB EPS ratio, and PN/PS ratio) and settling properties. Conversely, the introduction of an organic substrate (during the heterotrophic feeding regime) resulted in an abundance of r-strategist bacteria, and biomass washout in R1. The alpha diversity analyses show that the longer autotrophic enrichment phase in R1 resulted in the loss of microbial diversity that makes the biomass less resilient to the change in feeding regime. Optimization of the autotrophic and heterotrophic conditions to find a balance is desirable for the retention of nitrifying bacteria and the biological conversion of N_2_ in wastewater treatment systems. In this study, the abundance of denitrifying bacteria was observed during phase-two which shows a potential for an efficient simultaneous nitrification and denitrification in a system. A stepwise incremental increase of the organic substrate during phase-two, to gradually enrich the heterotrophic denitrifying bacteria able to use nitrate as an electron acceptor in place of dissolved oxygen may provide a path for optimizing a two-step process of biological nutrient removal.

## Conclusion

The paper describes unique features of both the physical–chemical characteristics of the microbial structures and the dynamics of dominant nitrifying bacteria in a system designed and operated to optimize autotrophic growth and to examine the influence of heterotrophic growth on nitrification. Comammox *Nitrospira* were the predominant ammonia-oxidizing bacteria in the seed in bioreactors and during treatment of relatively low influent ammonia concentrations. In response to increasing concentrations of ammonia, the relative abundance of AOB increased in relation to comammox *Nitrospira*. Optimizing for autotrophic conditions improved retention of the *Nitrospira* dominant pin-point flocs that exhibited granular sludge-like settling properties. The settling properties of biomass deteriorated in both bioreactors during the heterotrophic phase. Similarly, the PN:PS ratio in the EPS, and TB:LB EPS ratio gradually increased during the autotrophic phase, and declined during the heterotrophic phase. A relatively dominant autotrophic population enhances settling properties, whereas changing conditions that led to an increase in heterotrophic bacteria deteriorates the settling properties of the pin-point flocs.

## Data availability statement

The datasets presented in this study are deposited in the NCBI database under accession number PRJNA1023363.

## Author contributions

HA: Conceptualization, Data curation, Formal analysis, Investigation, Methodology, Project administration, Software, Validation, Visualization, Writing – original draft, Writing – review & editing. BA: Data curation, Methodology, Software, Writing – review & editing. SL: Conceptualization, Funding acquisition, Investigation, Resources, Supervision, Validation, Writing – review & editing.
