# Supplementary material for: Nitrospira dominant pin-point flocs with granule-like settleability in stirred tank reactors with oxic/hypoxic/oxic zones
Source: Front Microbiol. 2023 Dec 4;14:1307727. doi: 10.3389/fmicb.2023.1307727 (PMC10726125; doi:10.3389/fmicb.2023.1307727)
Supplement: Supplementary file 1 [file Data_Sheet_1.docx]

Supplementary Material

*Nitrospira* dominant pin-point flocs with granule-like settleability in stirred tank reactors with oxic/hypoxic/oxic zones

Hussain Aqeel^1^, Bruke Asefa^1^, Steven N Liss^1,2,3,*^

^1^Chemistry and Biology, Toronto Metropolitan University, Toronto, Ontario, Canada

^2^School of Environmental Studies, Queen’s University, Kingston, Ontario, Canada

^3^Department of Microbiology, Stellenbosch University, Stellenbosch, Western Cape, South Africa

*** Correspondence:**Prof. Steven N. Liss
[steven.liss@torontomu.ca](mailto:steven.liss@torontomu.ca)


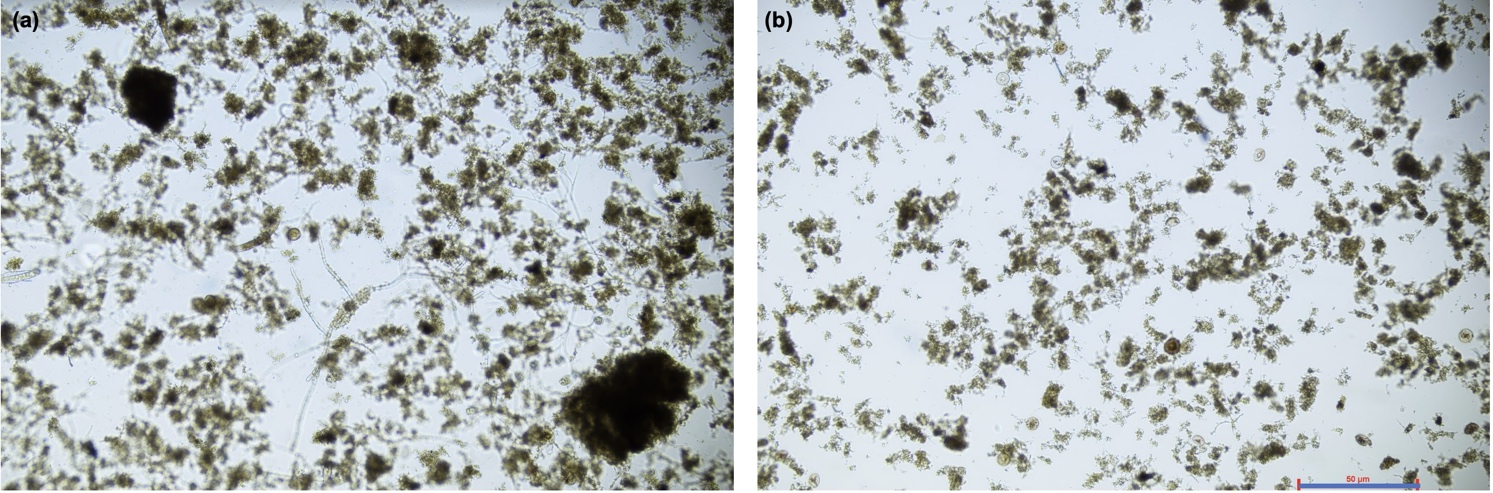


**Fig. S1** Optical microscope image showing the pin-point flocs of the seed biomass (a) and during the autotrophic phase (b) in the bioreactors. Scale bar = 50μm.

**
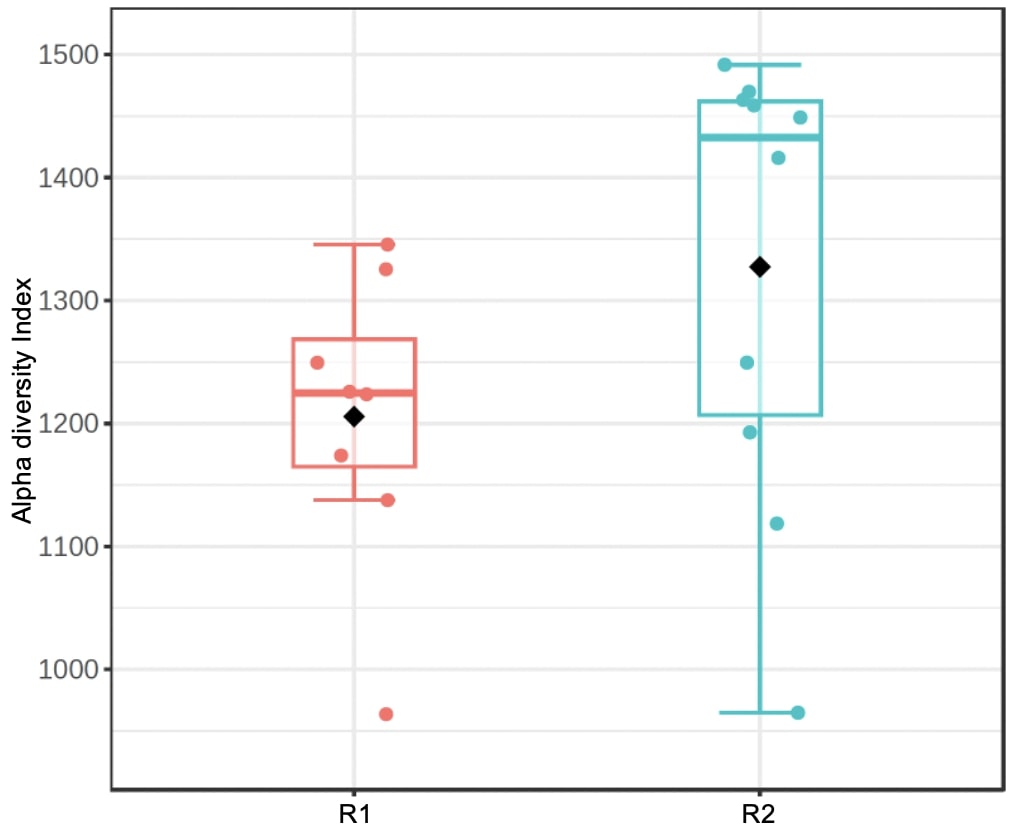
**

**Fig. S2** Alpha diversity index based on observed species metrics in R1 and R2.
